# Determinants of mortality among hospitalized patients with COVID-19 during first and second waves of the pandemic: A retrospective cohort study from an isolation center in Kano, Nigeria

**DOI:** 10.1371/journal.pone.0281455

**Published:** 2023-02-06

**Authors:** Farouq Muhammad Dayyab, Hussain Abdullahi Bashir, Abdulwahab Kabir Sulaiman, Garba Iliyasu, Muhammad Hamza, Ahmad Maifada Yakasai, Ibrahim Nashabaru, Hadiza Saidu, Bashir Garba Ahmad, Bashir Dabo, Aminu Yusuf Abubakar, Ibrahim Musa Idris, Abdulrauf Sani Yahaya, Mustapha Ado, Ibrahim Sabo Abdurrahman, Hafizu Musa Usman, Mohammed Kabiru Bello, Jaafar Suleiman Jaafar, Anifowose Abdullahi, Abubakar Muhammad Alhassan, Abdulmalik Ahmad, Alika Ehima Allen, Medu Oghenekevwe Ezekiel, Muhammad Abdullahi Umar, Muhammad B. Abdullahi, Sahabi Kabir Sulaiman, Tijjani Hussaini, Amina Abdullahi Umar, Aminu Ibrahim Tsanyawa, Sabitu Y. Shuaibu, Nasir Alhassan Kabo, Basheer Lawan Muhammad, Mohammed Nura Yahaya, Imam Wada Bello, Ashiru Rajab, Abdulhakim Muhammad Daiyab, Aminu Faruk Kabara, Muhammad Sule Garko, Abdulrazaq Garba Habib

**Affiliations:** 1 Department of Medicine, Infectious Disease Hospital, Kano, Kano State, Nigeria; 2 Department of Medicine, Kwanar Dawaki Isolation Center, Kano, Kano State, Nigeria; 3 Department of Medicine, Muhammad Abdullahi Wase Teaching Hospital, Kano, Kano State, Nigeria; 4 Department of Medicine, Murtala Muhammad Specialist Hospital, Kano, Kano State, Nigeria; 5 Infectious Disease Unit, Department of Medicine, College of Health Sciences, Bayero University, Kano, Kano State, Nigeria; 6 Department of Medicine, Yusuf Maitama Sule University, Kano, Kano State, Nigeria; 7 Cardiology Unit, Department of Medicine, College of Health Sciences, Bayero University, Kano, Kano State, Nigeria; 8 Department of Epidemiology, College of Public Health, University of South Florida, Tampa, Florida, United States of America; 9 Department of Medicine, Yobe State University Teaching Hospital, Yobe, Yobe State, Nigeria; 10 Kano State Primary Health Care Management Board, Kano, Kano State, Nigeria; 11 Division of Epidemiology and Biostatistics, Department of Community Medicine, Bayero University, Kano, Kano State, Nigeria; 12 Kano State Ministry of Health, Kano, Kano State, Nigeria; 13 Kano State Agency for Control of AIDS, Kano, Kano State, Nigeria; 14 Kano State Hospitals Management Board, Kano, Kano State, Nigeria; 15 Health Development Alternative Initiative, Kano, Kano State, Nigeria; 16 Clinton Health Access Initiative (CHAI), Lagos, Nigeria; 17 Department of Anesthesia, Muhammad Abdullahi Wase Teaching Hospital, Kano, Kano State, Nigeria; Lithuanian University of Health Sciences, LITHUANIA

## Abstract

**Background:**

Coronavirus disease 2019 (COVID-19) has emerged as an important cause of morbidity and mortality worldwide.

The aim of this study is to identify the clinical predictors of mortality among patients with COVID-19 pneumonia during first and second waves in a treatment center in northwestern Nigeria.

**Methods:**

This was a retrospective cohort study of 195 patients hospitalized with COVID-19 between April 2020 to March 2021 at a designated COVID-19 isolation center in Kano State, Northwest Nigeria. Data were summarized using frequencies and percentages. Unadjusted odds ratios and 95% confidence intervals and p-values were obtained. To determine independent determinants of mortality, we performed a stepwise multivariate logistic regression model.

**Results:**

Of 195 patients studied, 21(10.77%) patients died. Males comprised 158 (81.03%) of the study population. In the adjusted stepwise logistic regression analysis, age>64 years (OR = 9.476, 95% CI: 2.181–41.165), second wave of the pandemic (OR = 49.340, 95% CI:6.222–391.247), cardiac complications (OR = 24.984, 95% CI: 3.618–172.508), hypertension (OR = 5.831, 95% CI:1.413–24.065) and lowest systolic blood pressure while on admission greater than or equal to 90mmHg were independent predictors of mortality (OR = 0.111, 95%CI: 0.021–0.581).

**Conclusion:**

Strategies targeted to prioritize needed care to patients with identified factors that predict mortality might improve patient outcome.

## Introduction

Coronavirus Disease 2019, commonly referred to as COVID-19, caused by a novel coronavirus named Severe Acute Respiratory Syndrome Coronavirus-2 (SARS CoV-2) virus, first emerged in Wuhan City, Hubei Province, China in December 2019 [1]. The outbreak was declared as a Public Health Emergency of International Concern on January 30 2020 [1] and a pandemic in March 11 2020 [2] by the World Health Organization (WHO). COVID-19 was first reported in Nigeria on February 27, 2020 [3] when an Italian visitor tested positive for the virus. By April 11, 2020, the first patient confirmed to have the disease was diagnosed in Kano State and promptly isolated at the Kwanar Dawaki isolation center.

The pandemic is currently ongoing with over 500 million cases and over 6 million deaths worldwide as of May 1, 2022 [4]. Nationally, about 255,699 cases and about 3143 deaths were reported as of April 24, 2022 [5]. Furthermore, Kano State Nigeria reported about 4986 cases and about 126 deaths as of April 24, 2022 [5].

The investigation of factors that determine COVID-19 mortality is of paramount importance and will assist policy makers to make decisions that will halt the loss of lives associated with the disease. Hassan and colleagues reported that at the Nigerian population level, higher economic development is associated with mortality while lower death rates were reported in states with higher human immunodeficiency virus (HIV) prevalence and Bacillus Calmette-Guerin (BCG) vaccination coverage [6]. Osibogun et al reported on the comorbidities associated with mortality among 2184 patients with confirmed COVID-19 from Lagos, Southwestern Nigeria. They found that comorbidities that predicted death were hypertension, diabetes, renal disease, cancer and HIV [7]. Furthermore, a nationwide study by Elimian and colleagues reported that age greater than 51years, farming occupation, cough, vomiting and difficulty in breathing are associated with mortality among COVID-19 patients in Nigeria [8]. Another study in Nigeria by Akinbolagbe et al, showed that, age greater than 60 years, difficulty in breathing, and fever were independent predictors of hypoxaemia and death [9]. Similarly, in another study, hypoxaemia, obesity, diabetes mellitus and longer duration of symptoms were identified as predictors of mortality [10]. Cabo et al, reported the impact of demographic variables on COVID-19 mortality [11]. This study analysed the mean difference in the countries with a progressive and regressive population pyramid. It was reported that countries with progressive population pyramids present significantly less mortality than those with a regressive pyramid meaning that mortality was higher in countries with proportionally large numbers of aging population [11].

An epidemiological study in Nigeria reported higher number of tests carried out, less symptomatic cases, and less mortality during the second wave when compared to the first wave of the pandemic [12]. However, to date few studies in Nigeria evaluated the differences in clinical presentation and outcomes during the first and the second wave of the pandemic [10]. Our study differs from most of the studies described above as we included both the second and first waves, as opposed to just the latter.

The aim of this study is to identify the clinical predictors of mortality among patients with COVID-19 pneumonia during the first and the second waves in a treatment center in northwestern Nigeria.

## Methods

### Study design, setting and period

This was a retrospective cohort study of patients admitted with COVID-19 at Kwanar Dawaki isolation center in Kano. The cohort included all patients admitted for COVID-19 between April 2020 –March 2021. This period comprised of the first (April 2020 to October 2020) and second (November 2020 to March 2021) waves of COVID-19. The isolation center located in Dawakin Kudu, Kano, Nigeria, is a 79-bed COVID-19 care center. The hospital is one of the two centers dedicated to management of patients with COVID-19 in Kano State (with a projected population of 15 million based on the 2006 census). In addition to management of patients with mild to moderate disease, the Kwanar Dawaki isolation center was dedicated to management of patients with severe COVID-19 pneumonia in the state at the time of the study. The COVID-19 Care program is funded by the Kano State Government through the Kano State Task Force on COVID-19. Reverse Transcriptase-Polymerase Chain Reaction (RT-PCR) testing was used to investigate patients suspected to have clinical features suggestive of SARS-CoV-2 based on the Nigerian Center for Disease Control (NCDC) COVID-19 criteria [13]. Samples were collected and transported to laboratories certified by NCDC according to NCDC procedures [14]. All microbiological procedures were conducted according to WHO guidelines [15].

Newly diagnosed patients are transferred to the center and admitted immediately. This was then followed by testing of the patients contacts. Care is provided by a team of healthcare workers engaged on either permanent or part-time basis by the government.

### Study population and data collection

The COVID-19 treatment protocol for the Kwanar Dawaki isolation center was designed in line with modified NCDC guideline for management of COVID-19 [16]. Only patients who tested positive for SARS CoV-2 Polymerase Chain Reaction (PCR) were admitted at the center. All admitted patients are clinically evaluated and relevant laboratory investigations were conducted such as complete blood count, renal function tests, liver function tests, chest radiography, electrocardiography, and other relevant tests. Patients were categorized as having mild to moderate disease if they present with or without fever but, temperature is generally <38°C, with or without cough, do not have difficulty in breathing and have no underlying chronic heart or lung disease. Severe to life threatening category have difficulty in breathing, respiratory rate greater than 30 cycles per minute, high fever with temperature>38°C; or presence of underlying chronic medical conditions [16]. The guideline used in the management of the patients has undergone modifications. For instance, during the first wave of the pandemic, patients with mild-moderate disease were managed only symptomatically with vitamin C, zinc sulphate, paracetamol and loratadine, while patients with severe-life threatening disease in addition may receive azithromycin, hydroxychloroquine, oxygen, low molecular weight heparin, lopinavir, and corticosteroids. During the second wave of the pandemic, patients with mild-moderate disease were still managed symptomatically, however, patient with severe-life threatening disease no longer receive hydroxychloroquine and lopinavir but may also receive calcium supplements, and ivermectin in addition to those medications used during the first wave. This is a result of evolving evidence for-or-against repurposed medications evaluated in clinical trials. As at the time of the study, some life-saving procedures such as mechanical ventilation, hemodialysis were lacking at the center. Oxygen therapy was mainly delivered via nasal prongs, oxygen mask or re-breather bag.

Data for all COVID-19 patients that were ever admitted in the facility within the study period was retrospectively abstracted from patient health records by trained research assistants into Microsoft Excel (Version 2013; Microsoft Corporation, Redmond, WA, USA) spreadsheets, cleaned and analyzed using STATA SE version 13.0 (Stata Corp, College Station, Texas, USA) as detailed below.

The study sample comprised of 332 observations. We included all patients admitted at the isolation center during the first and second waves of the COVID-19 pandemic. The exclusion criteria were individuals with missing data for any of the independent variables. The final sample we analyzed included 195 patients.

### Description of variables

Our study determined association of the following independent variables with mortality: age in years (0–64, >64), sex (male, female), COVID-19 pandemic wave (first, second). Other independent variables included the clinical symptoms of cough, fever, shortness of breath (SOB), anosmia (Yes, No), disease severity (asymptomatic, mild to moderate, severe to life-threatening), lowest systolic blood pressure recorded in mmHg (<90, ≥90), lowest diastolic blood pressure recorded in mmHg (<60, ≥60), percent oxygen saturation on admission(<95, ≥95), complications and comorbidities such as acute kidney injury, sepsis, dyselectrolytaemia, respiratory failure, cardiac complication, hypertension, diabetes mellitus, bronchial asthma (Yes, No) and medications such as lopinavir, ceftriaxone, azithromycin, zinc, vitamin C, low molecular weight heparin, non-steroidal anti-inflammatory drugs(NSAIDS), corticosteroids, ivermectin(Yes, No). All were categorical variables. The primary outcome was whether an individual was discharged alive or dead (defined as COVID-19 attributable death based on a single chart reviewer).

### Handling missing data

**Fig 1:**
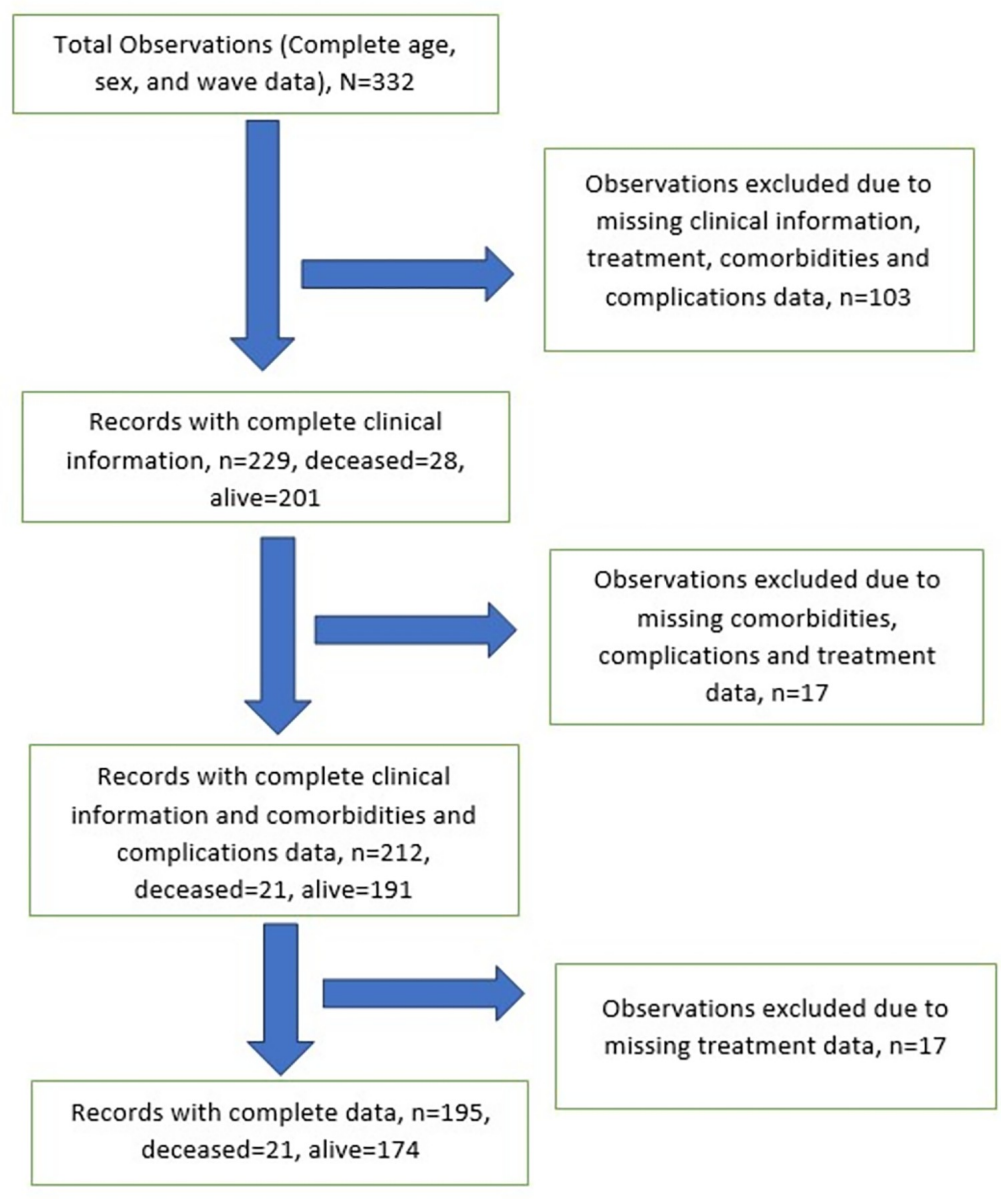
Flow diagram of patient selection (note that some observations have missing data on more than one variable).

All individuals in our database have information on their age, sex, the wave they were admitted and the outcome of the admission. Individuals without records on clinical presentation (fever, cough, shortness of breath, anosmia and disease category), complications and comorbidities (acute kidney injury, sepsis, dyselectrolytaemia, respiratory failure, cardiac complication, hypertension, diabetes mellitus, and bronchial asthma) and treatment (lopinavir, ceftriaxone, azithromycin, zinc, vitamin C, low molecular weight heparin, non-steroidal anti-inflammatory drugs, corticosteroids and ivermectin) were excluded from the analysis. Therefore, we used the complete-case approach for missing data handling. Fig 1 shows a flowchart that describes the final dataset selection processes for this study.

### Definition of key study variables

Acute kidney injury was defined based on Kidney Disease Improving Global Outcomes (KDIGO) criteria 12 as presence of any of the following [17]:

- Increase in serum creatinine by 0.3mg/dL or more within 48 hours or
- Increase in serum creatinine to 1.5 times baseline or more within the last 7 days or
- Urine output less than 0.5 mL/kg/h for 6 hours

Sepsis was defined based on the Systemic Inflammatory Response Syndrome (SIRS) criteria [18].

Hypertension was defined as systolic blood pressure of ≥140mmHg and diastolic blood pressure of ≥ 90mmHg based on WHO criteria [19].

Diabetes mellitus was defined as fasting blood sugar (FBS) of ≥7mmols/l or 2 hour postprandial value of ≥11.1mmols/l [20].

Cardiac complication in the study was considered as the presence of any form of arrhythmias detected by ECG, myocardial infarction, pulmonary embolism, or cardiac arrest [21].

### Data analysis

Data were summarized using frequencies and percentages. Unadjusted odds ratios and 95% confidence intervals and p-values were obtained using univariate logistic regression. To determine independent determinants of mortality, we performed a stepwise multivariate logistic regression model. All statistical analysis was done using STATA SE version 13.0 (Stata Corp, College Station, Texas, USA).

### Ethical approval

Ethical approval for the study was obtained from the Health Research Ethics Committee of Kano State Ministry of Health (MOH/Off/797/T. I/2042).

## Results

### Baseline characteristics and clinical data

As shown in Table 1, the study population included 195 patients confirmed to have COVID-19 based on a positive SARS CoV-2 PCR test with 21 deaths. The mean age of the study population was 45years and majority of the patients were in the age group 0–49 years. Majority of the respondents were males 158 (81.03%). At presentation, 33.33%, 37.95%, 23.56% and 3.08% of the patients had fever, cough, shortness of breath and anosmia respectively (Table 2). Majority of the patients were asymptomatic at presentation (60.51%). Hypertension (28.72%), diabetes mellitus (10.77%) and bronchial asthma (3.59%) were the commonest comorbidities among the patients (Table 3). The commonest complications suffered by the patients in our cohort were cardiac complications (9.23%), acute kidney injury (5.13%), sepsis (4.62%), dyselectrolytaemia (3.59%) and respiratory failure (3.59%). All the patients in the cohort received vitamin C as part of their treatment.

**Table 1:** Characteristics of study population and univariate analysis of mortality risk factors of COVID-19 patients (N = 195) at the Kwanar Dawaki isolation center, Kano, Nigeria.

Characteristics	Category	Total n (%)	Deceased n = 21	Survivors n = 174	Unadjusted Odds Ratio [95% CI; *p* value]
Age (years)	0–64	162(83.08)	10(47.62)	152(87.38)	Reference
	>64	33(16.92)	11(52.38)	22(12.64)	7.6[2.892–19.969, <0.0001]
Sex	Female	37(18.97)	0	37(21.26)	
	Male	158(81.03)	21(100)	137(78.74)	
Wave	1^st^ wave	98(50.26)	3(14.29)	95(54.60)	Reference
	2^nd^ wave	97(49.74)	18(85.71)	79(45.40)	7.215[2.050–25.389, 0.002]

**Table 2:** Clinical presentation of patients and univariate analysis of mortality risk factors of COVID-19 patients (N = 195) at Kwanar Dawaki isolation center, Kano, Nigeria.

Characteristics	Category	Total n (%)	Deceased n = 21	Survivors n = 174	Unadjusted Odds Ratio [95% CI; *p* value]
Fever	No	130 (66.67)	11(52.38)	130(66.67)	Reference
	Yes	65(33.33)	10(47.62)	65(33.33)	1.967 [0.789–4.906;0.147]
Cough	No	121(62.05)	12(57.14)	109(62.64)	Reference
	Yes	74(37.95)	9(42.86)	65(37.36)	1.258 [0.503–3.147;0.624]
SOB	No	149(76.41)	12(57.14)	137(78.74)	Reference
	Yes	46(23.56)	9(42.86)	37(21.26)	2.777 [1.088–7.091;0.033]
Anosmia	No	189(96.92)	20(95.24)	169(97.13)	Reference
	Yes	6(3.08)	1(4.76)	5(2.87)	1.690[0.188–15.199;0.640
Disease category	Asymptomatic	118(60.51)	7(33.33)	111(63.79)	Reference
	Mild to moderate	45(23.08)	8(38.1)	37(21.26)	3.429[1.164–10.1;0.025]
	Severe to Life threatening	32(16.41)	6(28.57)	26(14.94)	3.659[1.135–11.803;0.030]
Lowest SBP recorded	<90	20(10.26)	6(28.57)	14(8.05)	Reference
	≥90	175(89.74)	15(71.43)	160(91.95)	0.219[0.073–0.653;0.006]
Lowest DBP recorded	<60	30(15.38)	4(19.05)	26(14.94)	Reference
	≥60	165(84.62)	17(80.95)	148(85.06)	0.747[0.233–2.397;0.623]
SPO2 on admission	<95	47(24.10)	4(19.05)	43(24.71)	Reference
	≥95	148(75.90)	17(80.95)	131(75.29)	1.395[0.445–4.372;0.568]

SOB = Shortness of breath, SBP = Systolic blood pressure, DBP = Diastolic blood pressure, SPO2 = Oxygen saturation

**Table 3:** Comorbidities, complications and univariate analysis of mortality risk factors of COVID-19 patients (N = 195) at Kwanar Dawaki isolation center, Kano, Nigeria.

Characteristics	Category	Total n (%)	Deceased n = 21	Survivors n = 174	Unadjusted Odds Ratio [95% CI; *p* value]
Acute Kidney Injury	No	185(94.87)	18(85.71)	167(95.98)	Reference
	Yes	10(5.13)	3(14.29)	7(4.02)	3.976[0.945–16.736;0.060]
Sepsis	No	186(95.38)	18(85.71)	168(96.55)	Reference
	Yes	9(4.62)	3(14.29)	6(3.45)	4.667[1.074–20.270;0.040]
Dyselectrolytaemia	No	188(96.41)	18(85.71)	170(97.70)	Reference
	Yes	7(3.59)	3(14.29)	4(2.30)	7.083[1.468–34.177;0.015]
Respiratory failure	No	188(96.41)	18(85.71)	170(97.70)	Reference
	Yes	7(3.59)	3(14.29)	4(2.30)	7.083[1.468–34.177;0.015]
Cardiac complication	No	177(90.77)	14(66.67)	163(93.68)	Reference
	Yes	18(9.23)	7(33.33)	11(6.32)	7.409[2.482–22.116;0.000]
Hypertension	No	139(71.28)	11(52.38)	128(73.56)	Reference
	Yes	56(28.72)	10(47.62)	46(26.44)	2.530[1.008–6.349;0.048]
Diabetes Mellitus	No	174(89.23)	20(95.24)	154(88.51)	Reference
	Yes	21(10.77)	1(4.76)	20(11.49)	0.385[0.049–3.026;0.364]
Bronchial asthma	No	188(96.41)	20(95.24)	168(96.55)	Reference
	Yes	7(3.59)	1(4.76)	6(3.45)	1.4[0.160–12.227;0.761]

In our cohort, patients aged more than 64 years are more likely to die compared to those aged 0–49 years (p<0.0001) in the univariate analysis. There were no females among the deceased. Patients that presented with shortness of breath are more likely to be in the deceased group (p = 0.033) in the univariate analysis. In the univariate analysis, those with mild to moderate symptoms (p = 0.025) and those with severe to life-threatening symptoms (p = 0.030) are more likely to be in the deceased group compared to the asymptomatic patients. Patients whose lowest systolic blood pressure while on admission was greater than or equal to 90mmHg are less likely to be in the deceased group compared to those with less than 90mmHg (Table 2). Among the various comorbidities, only hypertension predicted mortality (p = 0.048) in the univariate analysis (Table 3). In the univariate analysis, acute kidney injury did not predict mortality (Table 3). None of the medications given to the patients showed a statistically significant mortality benefit (Table 4).

**Table 4:** Treatment and univariate analysis of mortality risk factors of COVID-19 patients (N = 195) at Kwanar Dawaki isolation center, Kano, Nigeria.

Characteristics	Category	Total n (%)	Deceased n = 21	Survivors n = 174	Unadjusted Odds Ratio [95% CI; *p* value]
Lopinavir	No	107(54.87)	13(61.90)	94(54.02)	Reference
	Yes	88(45.13)	8(38.10)	80(45.98)	0.723[0.285–1.832;0.494]
Ceftriaxone	No	138(70.77)	15(71.43)	123(70.69)	Reference
	Yes	57(29.23)	6(28.57)	51(29.31)	0.965[0.354–2.626;0.944]
Azithromycin	No	46(23.59)	2(9.52)	44(25.29)	Reference
	Yes	149(76.41)	19(90.48)	130(74.71)	3.215[0.720–14.361;0.126]
Zinc	No	2(1.03)	0	2(1.15)	
	Yes	193(98.97)	21(100.00)	172(98.85)	
Vitamin C	No	0	0	0	
	Yes	195(100.00)	21(100.00)	174(100.00)	
LMWH	No	138(70.77)	13(61.90)	125(71.84)	Reference
	Yes	57(29.23)	8(38.10)	49(28.16)	1.570[0.613–4.021;0.347]
NSAIDS	No	162(83.08)	21(100.00)	141(81.03)	
	Yes	33(16.92)	0	33(18.97)	
Corticosteroids	No	169(86.67)	21(100.00)	148(85.06)	
	Yes	26(13.33)	0	26(14.94)	
Ivermectin	No	175(89.74)	19(90.48)	156(89.66)	Reference
	Yes	20(10.26)	2(9.52)	18(10.34)	0.912[0.196–4.241;0.907]

LMWH = Low Molecular Weight Heparin, NSAIDS = Non-Steroidal Anti-inflammatory Drugs

Following the univariate analysis, variables with P values<0.25 were selected for inclusion in the model. Thirteen variables satisfied these criteria. Below is the fitted model.

Log (p/1-p) = β_0_ + β_1_age + β_6_lowestSBPrecorded + β_8_sepsis + β_2_wave + β_3_fever+ β_4_sob+ β_5_diseasecategory + β_7_AKI + β_9_dyselectrolytaemia+ β_10_respiratoryfailure+ β_11_cardiaccomplication + β_12_hypertension + β_13_azithromycin

Covariates whose P-value in the univariate model is less than 0.25 were imputed in a logistic regression model to explore the association between the independent and dependent variable.

### Determinants of mortality

As shown in Table 5, in the adjusted logistic regression analysis, age>64 years (OR = 9.476, 95% CI: 2.181–41.165), second wave of the pandemic (OR = 49.340, 95% CI:6.222–391.247), cardiac complications (OR = 24.984, 95% CI: 3.618–172.508), hypertension (OR = 5.831, 95% CI:1.413–24.065) and lowest systolic blood pressure while on admission greater than or equal to 90mmHg were independent predictors of mortality (OR = 0.111, 95%CI: 0.021–0.581). When disease category is dichotomized into severe disease Vs non-severe disease (OR = 0.302, 95% CI: 0.061–1.484), the level of significance of all the variables in the model did not change.

**Table 5:** Predictors of mortality in multivariate analysis (logistic regression); log likelihood ratio—26.832, pseudo R2 = 0.517.

Predictor	Category	Adjusted Odds Ratio [95% CI; *p* value]
Age	0–64	Reference
	>64	9.476[2.181–41.165;0.003]
Wave	First wave	Reference
	Second wave	49.340[6.222–391.247;<0.0001]
Disease Category	Asymptomatic	Reference
	Mild to Moderate	1.143[0.255–5.116;0.862]
	Severe to life threatening	0.327[0.053–2.020;0.229]
Lowest SBP	<90	Reference
	≥90	0.111[0.021–0.581;0.009]
Cardiac complication	No	Reference
	Yes	24.984[3.618–172.508;0.001]
Hypertension	No	Reference
	Yes	5.831[1.413–24.065;0.015]

SBP = Systolic Blood Pressure

The main effects model is therefore shown below:

Log (p/1-p) = β_0_ + β_1_age + β_2_wave + β_3_hypertention+ β_4_cardiaccomplication + β_5_diseasecategory + β_6_lowestSBPrecorded

The Hosmer-Lameshow test was used to check the goodness of fit of the final model. The P value obtained for this test was 0.4616; therefore, the model is a good fit. The area under the ROC curve was found to be 0.9030 which indicates that the model has ability to accurately discriminate between the two outcome categories (i.e., “survivor” or “deceased”).

## Discussion

In Kano State, the Kwanar Dawaki isolation center and the Muhammadu Buhari Specialist Hospital served as isolation centers for patients diagnosed to have COVID-19. Holding areas were established in several hospitals in the state. Based on the admission guidelines of the Kano State Task Force on COVID-19, all patients with severe disease are admitted at the former health facility. Therefore, it is not surprising that the mortality rate from our analysis (10.77%) was higher than the 4.3% reported in the literature [22]. Similarly, it was previously shown that, patients with COVID-19 that are being hospitalized in Nigeria have an increased risk of death [16].

The factors that determined mortality in our study included age>64 years, second wave of the pandemic, cardiac complications, hypertension, and lowest systolic blood pressure while on admission greater than or equal to 90mmHg.

Our findings are in keeping with several other studies. Elimian et al reported among others that age > 51 years is an independent factor associated with mortality [8]. Similarly, Akinbolagbe et al, and Ibrahim et al, in Nigeria also reported increased mortality among the aged population [9, 10]. Age has also been described as the most important variable in predicting COVID-19 mortality in an international study [22]. Majority of the respondents in our cohort were male 158 (81.03%) and though not statistically significant, there were more deaths among males compared to females. Male sex and age greater than 60 years predicted mortality among COVID-19 patients in Ohio [23]. Independent of age and comorbidities, male sex predicted mortality in The Netherlands cohort [24]. Several reasons have been highlighted for the higher severity of COVID-19 in men compared to women. These include higher levels of antiotensin-converting-enzyme-2 in men compared to women and differences in immunological responses influenced by hormonal differences in men and women [25]. Furthermore, behavioral factors such as cigarette smoking and alcohol consumption are more prevalent in men than women and could predispose to severe disease [26]. Another study suggested that gender differences in patterns and rates of contact may explain why men have more disease hence are disproportionately affected with severe infections and mortality [27].

Similar to our findings, the second wave of the pandemic is associated with higher in-hospital mortality compared to the first wave in a previous study in Nigeria by Akande et al [12]. Similar finding was also reported in Europe [28] and South Africa [29]. The South African study suggested that the excess mortality associated with the second wave might be related to the new lineage 501Y.V2. However, the predominant strain that accounted for the second wave in Nigeria was mostly delta variant including AY.36 and others [30]. In our study, similar to other parts of the world, there was skyrocketing illness and death due to the delta variant in the second wave compared to the first wave [30]. Elimian and colleagues from Nigeria reported fewer deaths during the second wave compared to the first wave of the pandemic [31]. However, the researchers suggested the interpretation of their findings with caution due to the limitations in the surveillance data used in their study [31]. It is not surprising that our findings differ from that of Elimian and colleagues due to differences in the design of the two studies.

As in previous reports [32–34], our study suggested the association between hypertension and mortality in COVID-19 patients. In another study of COVID-19 patients, higher mortality was reported among hypertensive patients not receiving antihypertensive therapy compared to hypertensive patients on antihypertensive therapy [35]. Furthermore, there was no difference in mortality between those on renin-antiotensin-aldosterone system (RAAS) inhibitors and those on non- RAAS inhibitors [35]. In Lagos Nigeria, hypertension, diabetes, renal disease, cancer and HIV predicted mortality [7]. It is suggested that the association of hypertension with endothelial dysfunction and renin angiotensin aldosterone system dysregulation may lead to progression of COVID-19 [36].

Our study reported association of cardiac injury with COVID -19 mortality. This finding is consistent with previous reports [37, 38]. Several mechanisms have been suggested regarding the association of COVID-19 and cardiac complications. COVID-19 has been associated with myocardial injury, myocarditis, dysrhythmias, venous thromboembolic events and acute heart failure [21]. Therefore, its pertinent to identify those patients with cardiac complications for possible improved therapeutic approaches that may avert mortality [39].

This study is limited by the exclusion of many study subjects from the analysis due to substantial missing data and therefore, small sample size. Another limitation is the inadequate laboratory data in the subjects therefore we could not determine their association with mortality. The study is also limited by the retrospective design, therefore many additionally useful variables that are not available in the dataset could not be obtained and included in the analysis. Another limitation is that the patients have received more than one of the listed medications given simultaneously and adjusting for effect modification or confounding was not done. Furthermore, the study is limited by the number of outcomes to variables in the multivariate model. Despite these limitations, our study has succeeded in reporting the determinants of mortality in Northwestern Nigeria, though our findings cannot be generalized.

In conclusion, strategies targeted to prioritize needed care to patients with identified factors that predict mortality might improve patient outcome.
